# Complete Genome Sequence of Pseudomonas aeruginosa Phage UF_RH6, Isolated from Human Lung

**DOI:** 10.1128/mra.00206-23

**Published:** 2023-05-30

**Authors:** Abdolrazagh Hashemi Shahraki, Majid Vahed, Shahla Masood, Mehdi Mirsaeidi

**Affiliations:** a Division of Pulmonary, Critical Care, and Sleep Medicine, College of Medicine-Jacksonville, University of Florida, Jacksonville, Florida, USA; b Department of Pathology and Laboratory Medicine, College of Medicine-Jacksonville, University of Florida, Jacksonville, Florida, USA; Portland State University

## Abstract

We report the genome sequence of a lytic phage named UF_RH6, which infects Pseudomonas aeruginosa. This phage was isolated from a respiratory secretion sample from a patient with pulmonary P. aeruginosa. UF_RH6 belongs to the family *Caudoviricetes* and the genus *Samunavirus*. Its genome is 94,715 bp in length and encodes 130 proteins.

## ANNOUNCEMENT

Bacteriophages have been identified in environments that are conducive to bacterial survival, such as soil, water, and wastewater, as well as animal and human tissues (1, 2). We obtained respiratory secretions from a 65-year-old male patient who was diagnosed with Pseudomonas aeruginosa pneumonia and was admitted to the University of Florida hospital (University of Florida Health, Jacksonville, FL, USA). The collection of human leftover samples for phage isolation was granted ethical approval by the University of Florida institutional review boards (approval number IRB202102636). A respiratory secretion sample (1 mL) was mixed with SM buffer (9 mL; Thermo Fisher Scientific, USA) and filtered through a 0.2-μm syringe filter. Then, 10 μL of filtered sample was added to 400 μL of P. aeruginosa (strain DJ06) cultured in brain heart infusion broth and was incubated at 37°C for 20 min. The phage was isolated using double-layer agar by incubating the plates at 37°C for 24 h (3). The phage was purified via single plaque isolation. DNA was extracted from phage lysate (5 mL) using the QIAamp MinElute Virus kit (Qiagen, USA). The Illumina Nextera XT library preparation kit was used for DNA library preparation, and sequencing was carried out using an Illumina NovaSeq 6000 system (paired-end 150-cycle mode). Bcl2fastq v2.20 (Illumina) was utilized to demultiplex reads, and Cutadapt v2.8 was used to remove sequencing adaptors and low-quality bases (4). Using the read mapper of the STAR package, P. aeruginosa DNA was removed from the data (5). The unmapped paired-end reads were then assembled using MetaWRAP v1.2.00 (6), and the resulting consensus sequences with lengths of >5,000 bp were evaluated by QUAST v5.0.2 (7). Centrifuge v1.04b was utilized to analyze the assembled consensus sequences (8). CheckV v1.01 was applied to evaluate the viral genome completeness and to identify closed genomes (9). The taxonomic identity of the virus was characterized by NCBI BLASTn (10). PhageTerm was used to determine the phage termini (11). VICTOR was used for phylogenetic analysis (12). GeneMarkS was used to identify open reading frames (ORFs) (13). The genome was annotated based on PHASTER (14) and BLASTp (10) results, and tRNA sequences were determined by tRNAscan-SE (15). ResFinder v4.0 (16) was used to detect virulence factors, and the Antibiotic Resistance Genes Database (ARDB) (17) was used to detect antibiotic resistance factors. We used default parameters for all software.

**FIG 1:**
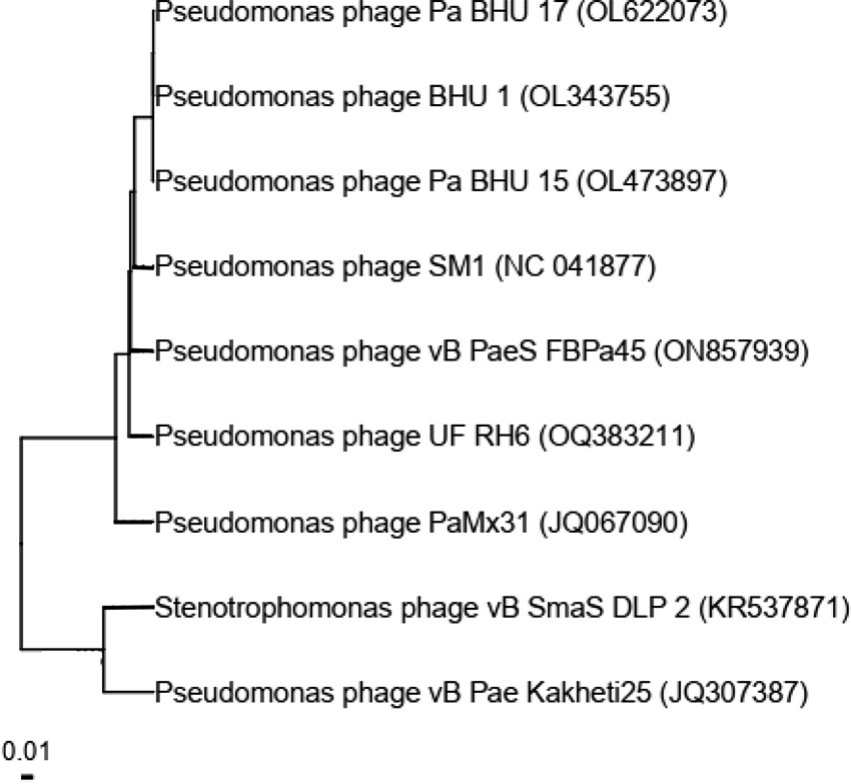
Phylogenetic tree illustrating the taxonomic relationship of UF_RH6 to closely related phages belonging to the genus *Samunavirus* (SM1, vB_PaeS_FBPa45, BHU-1, Pa BHU-15, Pa BHU-17, and PaMx31). Two other phages (*Stenotrophomonas* phage vB_SmaS_DLP_2 and Pseudomonas phage vB_Pae_Kakheti25) are Pseudomonas phages belonging to the genus *Septimatrevirus*. The tree was generated using the genome BLAST distance phylogeny (GBDP) method. The scale bar indicates the number of substitutions per site.

We obtained 24,108,049 raw reads (150-bp read length) for the sequenced sample. After removal of host DNA, only one contig with a length of >5,000 bp (94,715 bp) was assembled from the remaining reads (15.8% [3,809,071 reads]), with coverage of 10,768×. The genomic structure of Pseudomonas phage UF_RH6 (GenBank accession number OQ383211.1) is composed of linear double-stranded DNA, spanning a length of 94,715 bp and exhibiting a GC content of 55.30%. PhageTerm predicted a circularly permuted genome for UF_RH6. CheckV results showed the completeness of the sequence. The genome comprises 130 ORFs and belongs to the family *Caudoviricetes* and the genus *Samunavirus*, as evidenced by similarities to other members (Table 1 and Fig. 1). UF_RH6 shows the greatest nucleotide identity (99.08%) to Pseudomonas phage SM1 (GenBank accession number NC_041877.1). The genome of UF_RH6 contains a tRNA sequence; however, no virulence or antibiotic resistance genes were detected.

**TABLE 1:** Genome sequence coverage and nucleotide identity of UF_RH5 with respect to its closest relatives

Phage^*a*^	Sequence coverage (%)/identity (%) with respect to phage:					
	UF_RH6 (GenBank accession no. OQ383211.1)	SM1 (GenBank accession no. NC_041877.1)	vB_PaeS_FBPa45 (GenBank accession no. ON857939.1)	Pa BHU-15 (GenBank accession no. OL473897.1)	Pa BHU-17 (GenBank accession no. OL622073.1)	BHU-1 (GenBank accession no. OL343755.1)
UF_RH6 (GenBank accession no. OQ383211.1)	100/100	97/99.08	97/98.8	88/96.8	87/96.6	87/97.1
SM1 (GenBank accession no. NC_041877.1)	97/99.08	100/100	99/98.58	90/98.5	89/98.39	90/98.46
vB_PaeS_FBPa45 (GenBank accession no. ON857939.1)	97/98.8	99/98.58	100/100	90/98.1	89/98.39	90/98.99
Pa BHU-15 (GenBank accession no. OL473897.1)	88/96.8	90/98.5	90/98.1	100/100	93/100	93/100
Pa BHU-17 (GenBank accession no. OL622073.1)	87/96.6	89/98.39	89/98.39	93/100	100/100	93/100
BHU-1 (GenBank accession no. OL343755.1)	87/97.1	90/98.46	90/98.99	93/100	93/100	100/100

a All phages are classified as Pseudomonas phages.

### Data availability.

The complete phage genome sequence was deposited in GenBank under accession number OQ383211.1. The raw data are available in the NCBI Sequence Read Archive (SRA) under BioProject accession number PRJNA941099, SRA accession number SRR23702725, and BioSample accession number SAMN33589860.
